# Viridans streptococcus peritonitis in peritoneal dialysis: clinical characteristics and comparison with concurrent polymicrobial infection

**DOI:** 10.1186/s12882-018-1078-z

**Published:** 2018-10-19

**Authors:** Ying Liu, Ben-Chung Cheng, Jien-Wei Liu, Chiao-Jung Chen, Li-Chueh Kuo, Wen Xiu Chang, Jin-Bor Chen

**Affiliations:** 1 Department of Nephrology, Tianjin First Center Hospital, No.24 Fukang Road, Nankai District, Tianjin, 300192 China; 2 Division of Nephrology, Department of Internal Medicine, Kaohsiung Chang Gung Memorial Hospital, Chang Gung University College of Medicine, 123 Ta Pei Rd, Niao Song, District, Kaohsiung, 833 Taiwan; 3 Division of Infectious Diseases, Department of Internal Medicine, Kaohsiung Chang Gung Memorial Hospital, Chang Gung University College of Medicine, 123 Ta Pei Rd, Niao Song, District, Kaohsiung, 833 Taiwan

**Keywords:** *Viridans streptococci*, Peritonitis, Peritoneal dialysis

## Abstract

**Background:**

The clinical course of *Viridans streptococci* (VS) peritonitis in patients undergoing peritoneal dialysis (PD) is rarely reported. This study examined the association of clinical factors with VS peritonitis.

**Methods:**

We retrospectively reviewed clinical data from patients with VS peritonitis from March 1990 to February 2016 in a PD center in Taiwan and evaluated clinical profiles and treatment outcomes.

**Results:**

A total of 109 episodes of VS peritonitis in 71 patients identified. Among these patients, 57 had mono-VS peritonitis and 14 had concurrent polymicrobial infections. The median time interval from PD initiation to the first VS peritonitis episode was 18 months (range, 0.6–144 months). Among clinical outcomes, most VS peritonitis episodes were completely cured regardless of a history of peritonitis. All episodes with catheter removal occurred in those without a history of recent antibiotic use.

**Conclusion:**

VS peritonitis in patients undergoing PD typically has favorable treatment outcomes. Antibiotic therapy should be started promptly.

## Background

Viridans streptococci refer to a group of Streptococcus species that are nutritionally fastidious and mainly alpha-hemolytic on sheep blood agar; these gram-positive cocci are commensals of the oral cavity, upper airway, and the gastrointestinal and genitourinary tracts [1]. The major portals of entry for VS are damaged epithelial barriers of a patient’s mucosa.

Peritoneal dialysis (PD)-related peritonitis is a common complication in patients undergoing regular PD. Clinical courses of PD-related peritonitis vary from mild to severe forms, depending on the causative organisms and clinical conditions. The remarkable consequences of PD-related peritonitis are technique failure leading to PD catheter removal [2, 3]. Although gram-negative organisms have been increasingly detected in PD-related peritonitis in recent years, gram-positive organisms remain major pathogens for this infection entity [4–6]. Gram-positive cocci constitute around 43–64% of all PD peritonitis episodes, and Streptococcal species account for about 10–15% of them [7]. VS are the majority of Streptococcus species. The incidence of VS peritonitis in several reports from different regions was similar. The entry pathway of VS into the peritoneal cavity includes contamination during the exchange procedure, gastrointestinal bacterial translocation, haematological dissemination with oral and dental procedures and catheter related [7]. Of note, among PD-related peritonitis cases caused by gram-positive cocci, those caused by viridans streptococcus (VS) were reported to have a lower risk of PD catheter removal and chances of relapsing episodes [7] but a higher incidence of clinically refractory course [7].

Although mono-VS peritonitis in patients undergoing PD in general has a favorable outcome, little is known about the clinical outcomes of VS-included polymicrobial peritonitis (mixed-VS peritonitis) in this patient population. The aim of this retrospective study was to better understand the differences between mono-VS peritonitis and mixed-VS peritonitis in patients undergoing PD.

## Methods

### Study population

Patients undergoing PD at Kaohsiung Chang Gung Memorial Hospital in Taiwan between March 1990 and February 2016 were eligible for screening. Patients with PD-related peritonitis episodes in which VS, regardless of additional bacterial species, grew from the dialysate were included for analysis. Their medical notes were reviewed to retrieve the following information: demographics, primary kidney diseases, estimated glomerular filtration rate (eGFR), baseline body mass index, time elapsed from PD initiation to the first onset of PD-related peritonitis, recent antibiotic exposure (≦2 weeks) and prior peritonitis, and antibiotic(s) to which the culprit VS and other culprit bacteria were susceptible. eGFR was calculated based on urine collected for 24 h, and was normalized to body surface area using the Du Bois formula and patient body weight [8].

### Diagnosis and definitions of PD-related peritonitis

The diagnosis of PD-related peritonitis was made based on the findings of two or more of the following criteria: (1) abdominal pain or cloudy peritoneal dialysis effluent, (2) leukocytosis in peritoneal dialysis effluent (white cell count > 100/mL with > 50% polymorphonuclear leukocytes), and (3) positive gram stain or bacterial culture from peritoneal dialysis effluent [9]. Relapse PD-related peritonitis referred to one that occurred less than four weeks after the completion of antibiotic therapy of a prior PD-related peritonitis with the same pathogen [10]. Cured PD-related peritonitis was defined as the resolution of peritonitis by antibiotic therapy alone, without relapse or recurrence [11]. Peritonitis-related death referred to a fatality within two weeks involving a patient with active PD-related peritonitis [10]. Treatment failure referred to the discontinuation of either a temporary or a permanent PD and/or death due to PD-related peritonitis [12, 13].

### Bacterial identification and susceptibility testing

All bacterial isolates grown from peritoneal dialysate were identified on clinical-practice basis using conventional methods as described elsewhere [14–16] and were confirmed using the Phoenix automated microbiology system (Becton Dickinson Diagnostic Systems, Sparks, MD). Viridans streptococci were identified mainly based on the findings of non-ß-hemolytic gram-positive cocci in chain that were negative for catalase test, resistant to optochin, non-bile soluble, and negative for pyrrolidonyl arylamidase test [14]. Antibiotic susceptibility tests were also performed on a clinical-practice basis using the disk diffusion methods as recommended by the CLSI (previously known as the NCCLS) [17, 18]. Susceptibility testing for bacteria isolated from normally sterile sites including blood and dialysate was performed using microdilution methods (Phoenix automated microbiology system) according to the manufacturer’s instructions while testing for bacteria isolated from other sites was carried out using disk diffusion methods as recommended by the CLSI. The susceptible (S), intermediate (I), and resistant (R) categories were based on the criteria for the antibiotic susceptibility testing recommended by the CLSI guidelines issued in 2002 [19]. Antibiotics tested against VS isolates and the criteria for the diameters of the antibiotic inhibitory zones (mm) in disk diffusion methods were as follows: penicillin (S ≧ 24), ampicillin (S ≧ 24), ceftriaxone (S ≧ 27, *I* = 25–26, *R* < 24), vancomycin (*R* > 17), erythromycin (S ≧ 21, *I* = 16–20, *R* < 15), and clindamycin (S ≧ 19, I = 16–18, R < 15). Moreover, antibiotics tested against VS isolates and antibiotic breakpoints (μg/mL) in microdilution methods were as follows: penicillin (S ≦ 0.12, *I* = 0.25–2, R ≧ 4), ampicillin (S ≦ 0.25, *I* = 0.5–4, R ≧ 8), ceftriaxone (S ≦ 1, I = 2, R ≧ 4), vancomycin (S ≦ 1), erythromycin (S ≦ 0.25, I = 0.5, R ≧ 1), and clindamycin (S ≦ 0.25, I = 0.5, R ≧ 1) [19, 20]. Intermediate and resistance results in susceptibility testing were grouped as non-susceptibility. These cutoff criteria were adopted over the study period.

### Management of PD-related peritonitis

Antibiotic administration via the intraperitoneal route was initiated once patients were diagnosed with PD-related peritonitis. Empirical antibiotic treatment was based on recommendations by the International Society of Peritoneal Dialysis (ISPD). Specifically, cefazolin and gentamicin were empirically chosen as empirical antibiotics before the year 2000 [9, 21]; cefazolin and ceftazidime were empirically used in the following years [10, 22, 23]. These empirical antibiotics were subsequently adjusted based on the isolated pathogen(s) and antimicrobial susceptibility testing as necessary. In general, antimicrobial treatment lasted for at least two weeks, and the treatment duration might be extended based on the clinical judgment made by the patient’s attending nephrologist. PD catheters were removed if antimicrobial therapy alone failed to resolve the peritonitis within 2–3 weeks, and PD was then replaced by temporary or permanent hemodialysis.

This study was conducted with a waiver of patient consent approved by the Institutional Review Board of Chang Gung Memorial Hospital (Document no. 100-2661B).

### Statistical analysis

Demographic, clinical, and laboratory data between the mono-VS peritonitis and mixed-VS peritonitis groups were compared with each other. The Mann-Whitney U test was used for comparing continuous variables while Fisher’s exact test or chi-square test was used for comparing dichotomous variables. Two-tailed *P* values less than 0.05 were considered statistically significant. All statistical analyses were carried out using SPSS software (IBM SPSS Statistics for Windows, Version 20.0, IBM Corp. Armonk, NY).

## Results

A total of 109 VS peritonitis episodes were identified in 71 patients (57 with mono-VS peritonitis and 14 with mixed-VS peritonitis), accounting for 10.27% (109/1061) of the overall episodes of PD-related peritonitis during the study period. The frequency of peritonitis in our hospital is 0.20–0.27episodes/person/year, and VS peritonitis is 0.41episodes/person/year. In 38 episodes, patients’ blood samples were collected for culture; only *Cutibacterium acnes* grew in the blood drawn from 2 patients with mono-VS peritonitis, which were considered contaminants. Among the overall included patients, 1 episode was found in 51 patients, and repeated PD-related peritonitis episodes were found in the rest (specifically, 2 episodes in 12 patients, 3 in 3, 4 and 6 each in 2, and 5 in 1). Underlying diseases leading to end-stage renal disease, in descending order, were glomerulonephritis (50/71 [70.4%]), diabetic nephropathy (9/71 [12.8%]), hypertensive nephropathy (4/71 [5.6%]), lupus nephropathy (5/71 [7.0%]), and unknown (3/71 [4.0%]).

Of the overall included patients, the median age was 56 years (range, 16–81 years), the median time interval from PD initiation to the first VS peritonitis episode was 18 months (range, 0.6–144 months), and 40.8% of the VS peritonitis episodes occurred in more than 24 months after PD initiation. Between patients with mono-VS peritonitis and mixed-VS peritonitis, there were no difference in sex, age, underlying diabetes mellitus, albumin, eGFR, body mass index, or prior antibiotic exposure (Table 1). No patient was undergoing immunosuppressive therapy.

**Table 1:** Demographic and clinical features of patients suffering the first episode of PD related VS peritonitis

	Overall VS peritonitis patients (*N* = 71)	mono-VS peritonitis (*N* = 57)	mixed-VS peritonitis (*N* = 14)	*P* ^*^
Male (%)	30 (42.3%)	24 (42.1%)	6 (42.9%)	0.959
Age (years) (median, range)	56 (16–81)	56 (33–81)	55.5 (16–72)	0.275
First peritonitis after starting PD (months)				
Median, range	18.0 (0.6–144.0)	18.0 (0.6–144.0)	16.1(2.3–105.2)	0.919
Time interval				
< 6	13 (18.3%)	11 (19.3%)	2 (14.3%)	
6–12	12 (16.9%)	7 (12.3%)	5 (35.7%)	
12–24	17 (23.9%)	16 (28.1%)	1 (7.1%)	
> 24	29 (40.8%)	23 (40.4%)	6 (42.9%)	
Diabetes mellitus, *n* (%)	9 (12.7%)	9 (15.8%)	0 (0%)	0.112
Baseline albumin (gm/dl) (mean ± SD)	3.46 ± 0.51	3.46 ± 0.49	3.48 ± 0.62	0.900
Baseline eGFR (ml/min/1.73m^2^) (median, range)	6 (0–61)	6 (0–61)	11.5 (0–27)	0.406
Baseline BMI (kg/m^2^) (median, range)	22.5 (16.2–34.8)	22.7 (16.2–34.8)	21.7 (17.3–26.8)	0.333
Prior antibiotic exposure, n (%)	8 (11.3%)	8 (14%)	0 (0%)	0.155

Abbreviations: *VS* viridans streptococcus, *PD* peritoneal dialysis, *eGFR* estimated glomerular filtration rate, *BMI* body mass index

*Mono-VS peritonitis vs. mixed-VS peritonitis

Regarding the additional organisms other than VS found among the mixed-VS peritonitis episodes, 1 organism was found in 26 episodes, 2 in 4, and 3 in 1. The concurrent infectious organisms are shown in Table 2.

**Table 2:** Organisms other than viridans streptococcus (VS) isolated from 31 episodes of mixed-VS peritonitis

Organism	No. of isolates
Gram-positive cocci	
Coagulase-negative Staphylococci	2
*Staphylococcus aureus*	3
*Enterococcus* spp.	4
*Stomatococcus* spp*.*	2
Group B streptococcus	1
Gram-negative bacilli	
*Escherichia coli*	6
*Klebsiella oxytoca*	2
*Klebsiella pneumoniae*	8
*Acinetobacter baumannii*	1
*Enterobacter cloacae*	2
*Providencia stuartii*	1
*Pseudomonas* sp.	1
*Others*	
Gram-positive bacillus	1
*Neisseria* spp.	2
Fungus	
*Candida parapsilosis*	1

Of the VS isolates in mono-VS peritonitis episodes, 93.6% were susceptible to ampicillin, 91.0% to penicillin, and 91.2% to ceftriaxone. In contrast, of the VS isolates in mixed-VS peritonitis episodes, 90.3% were susceptible to ampicillin, 87.1% to penicillin, and 87.1% to ceftriaxone (Table 3). All viridans streptococci, regardless of being isolated from mono-VS or mixed-VS peritonitis patients, were susceptible to vancomycin and/or teicoplanin (Table 3). We also examined the association of antibiotic susceptibility with recent antibiotic exposure and prior peritonitis. In mono-VS peritonitis, the rate of those with recent antibiotic exposure was lower (13.0–16.7%) than those with prior peritonitis (47.4–52.7%) among susceptible episodes. The trend was similar in mixed-VS peritonitis wherein the rate of those with recent antibiotic exposure was lower (10.7–13.8%) than those with prior peritonitis (66.7–75.9%) among susceptible episodes. A detailed antibiotic susceptibility profile is shown in Table 4.

**Table 3:** Antibiotic susceptibility rates of VS isolates from VS peritonitis episodes in patients undergoing peritoneal dialysis

Tested antibiotic	VS isolates in mono-VS peritonitis(n)				VS isolates in mixed-VS peritonitis(n)			
	Susceptible (A)	Non-susceptible (B)	Susceptibility rate (%)	Non-susceptibility rate (%)	Susceptible (A)	Non-susceptible (B)	Susceptibility rate (%)	Non-susceptibility rate (%)
Ampicillin	73	5	93.6	6.4	28	3	90.3	9.7
Clindamycin	67	11	85.9	14.1	29	2	93.5	6.5
Ceftriaxone	62	6	91.2	8.8	27	4	87.1	12.9
Erythromycin	57	21	73.1	26.9	26	5	83.9	16.1
Penicillin	71	7	91.0	9.0	27	4	87.1	12.9
Teicoplanin	74	0	100	0	29	0	100	0
Vancomycin	66	0	100	0	31	0	100	0

Susceptibility rate = A/(A + B)

Non-susceptibility rate = B/(A + B)

**Table 4:** Antibiotic susceptibility profiles of VS isolates from VS peritonitis episodes in patients undergoing peritoneal dialysis

Tested antibiotic	VS isolates in mono-VS peritonitis, no./No. (%)				VS isolates in mixed-VS peritonitis, no./No. (%)				*P*					
	Susceptible		Non-susceptible		Susceptible		Non-susceptible							
	Recent antibiotic exposure (A)	Prior peritonitis (B)	Recent antibiotic exposure (C)	Prior peritonitis (D)	Recent antibiotic exposure (E)	Prior peritonitis (F)	Recent antibiotic exposure (G)	Prior peritonitis (H)	A vs. C	B vs. D	E vs. G	F vs. H	A vs. E	B vs. F
Ampicillin	11/73 (15.1)	37/73 (50.7)	1/5 (20.0)	3/5 (60.0)	3/28 (10.7)	19/28 (67.9)	1/3 (33.3)	3/3 (100.0)	0.577	1.000	0.349	0.537	0.806	0.120
Clindamycin	10/67 (15.0)	34/67 (50.7)	2/11 (18.2)	6/11 (54.5)	4/29 (13.8)	20/29 (69.0)	0/2 (0.0)	2/2 (100.0)	1.000	0.815	1.000	1.000	1.000	0.098
Ceftriaxone	8/62 (13.0)	30/62 (48.4)	2/6 (33.3)	4/6 (66.7)	1/27 (3.7)	18/27 (66.7)	3/4 (75.0)	4/4 (100.0)	0.212	0.669	0.003	0.295	0.347	0.112
Erythromycin	8/57 (14.0)	27/57 (47.4)	4/21 (19.0)	13/21 (61.9)	3/26 (11.5)	18/26 (69.2)	1/5 (20.0)	4/5 (80.0)	0.849	0.255	0.525	1.000	1.000	0.064
Penicillin	10/71 (14.1)	36/71 (50.7)	2/7 (28.6)	4/7 (57.1)	3/27 (11.1)	18/27 (66.7)	1/4 (25.0)	4/4 (100.0)	0.642	1.000	0.442	0.435	0.957	0.156
Teicoplanin	12/74 (16.2)	39/74 (52.7)	0/0 (0.0)	0/0 (0.0)	4/29 (13.8)	22/29 (75.9)	0/0 (0.0)	0/0 (0.0)	–	–	–	–	0.998	0.031
Vancomycin	11/66 (16.7)	33/66 (50.0)	0/0 (0.0)	0/0 (0.0)	4/31 (12.9)	22/31 (71.0)	0/0 (0.0)	0/0 (0.0)	–	–	–	–	0.860	0.052

no./No. = number of VS isolate(s) with recent antibiotic exposure or prior PD-peritonitis/number of VS isolates subject to antibiotic susceptibility testing

**Fig. 1:**
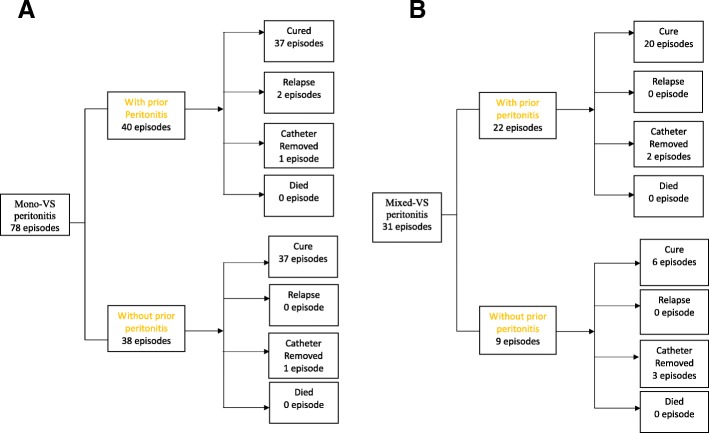
Summary of clinical outcomes of patients in episodes of (**a**) mono-VS peritonitis, and (**b**) mixed-VS peritonitis

The clinical outcomes are shown in Fig. 1. Among 78 episodes of mono-VS peritonitis, 40 involved patients with prior peritonitis wherein the majority were cured (37/40), while 38 involved patients with no prior peritonitis wherein 37 were cured (*P* = 0.744). Additionally, among 31 episodes of mixed-VS peritonitis, 22 included patients with prior peritonitis wherein 20 were cured, and 9 included patients with no prior peritonitis wherein 6 were cured (*P* = 0.259). There was no fatality in either group.

Table 5 presents the associations between clinical outcomes and recent antibiotic use. In mono-VS peritonitis, 11 episodes with recent antibiotic use were among 74 totally cured episodes. In two episodes with catheter removal, no episode occurred with recent antibiotic use. Overall, there was no significant difference in clinical outcome regardless of recent antibiotic use (*p* = 0.495). A total of 31 episodes of mixed-VS peritonitis were identified. Among the cured episodes, 4 episodes had recent antibiotic use. There was no relapse in the 31 episodes. All episodes with catheter removal did not have recent antibiotic use. There was no death in the study participants.

**Table 5:** Associations between clinical outcomes and recent antibiotic history

mono-VS peritonitis (*n* = 78 episodes)			
	Cure (*n* = 74)	Relapse (*n* = 2)	Catheter Removed (n = 2)
No recent antibiotic use	63	1	2
Recent antibiotic use	11	1	0
mixed-VS peritonitis (*n* = 31 episodes)			
	Cure (*n* = 26)	Relapse (*n* = 0)	Catheter Removed (n = 5)
No recent antibiotic use	22	0	5
Recent antibiotic use	4	0	0

Abbreviation: *VS* viridans streptococcus

## Discussion

With the inclusion of patients over a 26-year period in a large medical center, this is the largest cohort of patients undergoing PD with VS peritonitis. The baseline characteristics in the first episode of VS peritonitis revealed a predominance of female patients, and most first VS-episodes occurred in more than 24 months after PD initiation. A higher incidence of streptococcal peritonitis in women has been reported previously [7, 24], similar to the finding in this study. However, one report did not corroborate this finding [25]. One possible explanation for this difference in findings was that enterococcal peritonitis was included in previous reports. Enterococci are present in the female genital tract and perineal skin. Therefore, the likelihood of concurrent infection is higher in female patients. However, enterococci have been recognized as a separate genus of gram-positive cocci, which may have contributed to differences in reporting in recent years. Thus, further study is needed to fully examine this relationship.

Our data showed that majority of the first episodes of VS peritonitis occurred in more than 24 months after PD initiation with four time intervals. This time interval is similar in mono- and mixed-VS peritonitis episodes in the present study. Review of literature and prior studies reported the time interval from PD initiation to the first VS peritonitis episode by using mean or median values. A report from Canada and Taiwan on VS peritonitis in patients undergoing PD demonstrated the mean time interval to be more than 24 months for the first VS peritonitis episode [7, 25]. Moreover, this time interval is longer than that for *Escherichia coli* peritonitis (4.2–24.7 months, median 13.9 months) [13]. Differences in patient characteristics, patient management in individual PD centers, geographic locations, and reported time-interval methods may contribute to disparities in reported time intervals. Nevertheless, the results of the present study provide new data for peritonitis prevention in patients undergoing PD and emphasize the possible relationship between individual causative organisms of peritonitis and time elapsed since PD initiation.

With regard to antibiotic susceptibility, our data showed higher susceptibility rates in ampicillin, penicillin, and vancomycin. This finding is concordant with the ISPD guidelines, which recommend ampicillin as the first choice of treatment for VS peritonitis [10, 22]. History of antibiotic use and peritonitis could influence the susceptibility to antibiotics [13, 26]. The present study demonstrated that susceptibility rates for antibiotics were lower among those episodes with recent antibiotic exposure than in those with prior peritonitis. The trend was similar in the episodes in either mono-VS or mixed-VS peritonitis. Overall, our cohort had few non-susceptibility episodes. In these episodes, vancomycin and teicoplanin did not show non-susceptibility in either mono-VS peritonitis or mixed-VS peritonitis. The findings are compatible with the common knowledge that VS are susceptible to both antibiotics.

Noteworthy, favorable clinical outcomes were found in the involved patients, irrespective of having mono-VS peritonitis or mixed-VS peritonitis, and these patients had a lower PD catheter removal rate and peritonitis relapse rate compared to those reported in other series [7, 25]. The exact mechanism of this difference cannot be determined from information available in the literature. Nevertheless, the small number of reported instances of PD catheter removal and peritonitis relapse implies that these events are unusual among patients undergoing PD who develop VS peritonitis. In addition, our results suggested that peritonitis was possibly not timely detected at its early stage in the previous studies and clinicians should be alert to any clinical clue suggesting possible peritonitis in patients undergoing PD so that antibiotic therapy can be started promptly.

Although the present study provides some clinical information on VS peritonitis in patients undergoing PD, some limitations must be addressed. First, this study was a retrospective and single-center experience with patients undergoing PD with VS peritonitis. Second, we did not analyze the responses to the empirical and subsequent antibiotic treatments. To our knowledge, this has been the first study to evaluate the association between recent antibiotic use and technique outcome in VS peritonitis in patients undergoing PD. The clinical data were drawn in one PD center only; therefore, the heterogeneity in clinical care can be avoided as possible. A real world in VS peritonitis in patients undergoing PD could be realized in the present study.

## Conclusions

Generally, patients with VS peritonitis had a favorable clinical outcome. Compared with mono-VS peritonitis, the clinical characteristics of concurrent polymicrobial peritonitis are similar. To minimize the chances of PD catheter removal and relapse of peritonitis, clinicians should be alert to any clue suggesting possible early peritonitis so that empirical antibiotic therapy can be started promptly.

## Abbreviations

CLSI: Clinical and Laboratory Standards Institute
eGFR: Estimated glomerular filtration rate
I: Intermediate
ISPD: International Society of Peritoneal Dialysis
NCCLS: National Committee for Clinical Laboratory Standards
PD: Peritoneal dialysis
R: Resistant
S: Susceptible
VS: Viridans streptococcus
